# The MIL-88A-Derived Fe_3_O_4_-Carbon Hierarchical Nanocomposites for Electrochemical Sensing

**DOI:** 10.1038/srep14341

**Published:** 2015-09-21

**Authors:** Li Wang, Yayun Zhang, Xia Li, Yingzhen Xie, Juan He, Jie Yu, Yonghai Song

**Affiliations:** 1 Key Laboratory of Functional Small Organic Molecule, Ministry of Education, College of Chemistry and Chemical Engineering, Jiangxi Normal University, 99 Ziyang Road, Nanchang 330022, People’s Republic of China

## Abstract

Metal or metal oxides/carbon nanocomposites with hierarchical superstructures have become one of the most promising functional materials in sensor, catalysis, energy conversion, etc. In this work, novel hierarchical Fe_3_O_4_/carbon superstructures have been fabricated based on metal-organic frameworks (MOFs)-derived method. Three kinds of Fe-MOFs (MIL-88A) with different morphologies were prepared beforehand as templates, and then pyrolyzed to fabricate the corresponding novel hierarchical Fe_3_O_4_/carbon superstructures. The systematic studies on the thermal decomposition process of the three kinds of MIL-88A and the effect of template morphology on the products were carried out in detail. Scanning electron microscopy, transmission electron microscopy, X-ray powder diffraction, X-ray photoelectron spectroscopy and thermal analysis were employed to investigate the hierarchical Fe_3_O_4_/carbon superstructures. Based on these resulted hierarchical Fe_3_O_4_/carbon superstructures, a novel and sensitive nonenzymatic N-acetyl cysteine sensor was developed. The porous and hierarchical superstructures and large surface area of the as-formed Fe_3_O_4_/carbon superstructures eventually contributed to the good electrocatalytic activity of the prepared sensor towards the oxidation of N-acetyl cysteine. The proposed preparation method of the hierarchical Fe_3_O_4_/carbon superstructures is simple, efficient, cheap and easy to mass production. It might open up a new way for hierarchical superstructures preparation.

Fe_3_O_4_ has attracted tremendous attention for its novel magnetic and catalytic properties. However, its poor conductivity, easy aggregation and uselessness in strong acidic solution exclude it as promising materials in many fields such as electrochemistry and biology. To overcome these drawbacks, other agents (e.g., liposome, micelle, polymer, silica) with compensatory properties was introduced into Fe_3_O_4_^1^. Among them, carbon was the typical material used to promote Fe_3_O_4_’s conductivity and stability. For example, Fe_3_O_4_ embedded into porous carbon nanosheets or nanotube was benefit from the conductivity of carbon and used as a durable high-rate lithium ion battery anode material. At the same time, the carbon matrix might effectively inhibit the aggregation of Fe_3_O_4_^2^,^3^. The Fe_3_O_4_@carbon nanocomposites after further modification with strong oxidizing agents might be biocompatible and applied as drug delivery^4^. Recently, one-step hydrothermal synthesis of Fe_3_O_4_@carbon nanocomposites has been reported with great performance in biomedicine^5^.

Generally, there are two strategies to synthesize Fe_3_O_4_@carbon nanocomposites. The first method is wet chemistry, that is, Fe_3_O_4_@carbon is synthesized by mixing Fe_3_O_4_ nanoparticles or their precursors with a carbon source (e.g., glucose, dopamine, ethylene glycol, citric acid, oleic acid, EDTA, etc.) followed by a carbonization process^5^,^6^,^7^,^8^,^9^,^10^. For this strategy, a strong dependence on reaction conditions was required, thus aggregation and chemical wastes were inevitably occurred. Furthermore, most products had compact and smooth exteriors, limiting the effective utilization of inner surface. The second method is dry method such as magnetron sputtering. With this method, the resulted Fe_3_O_4_@carbon always showed low dimensionality^2^. In fact, the property of materials can be enhanced by tailoring their shapes, sizes and compositions^11^. Much effort has been devoted to design the morphology of materials for further promoting their performance^12^,^13^. Recently three-dimensional (3D) architecture was employed as a template to afford both high porosity and good conductivity^14^,^15^. For example, Pt-based bimetallic flower-like or dendritic-like NPs showed great potential as catalysts for reducing the Pt consumption, providing a high surface area, and facilitating enhanced performance in the catalytic applications^16^,^17^,^18^,^19^,^20^.

Recently, metal-organic framework (MOF), a new class of hybrid functional materials has attracted extensive attention for their diverse structures, topologies and compositions. The MOFs-template method has been adopted to form metal/metal oxide micro/nanostructures with various controlled shapes including microplates, nanowires, nanorods, nanoparticles, nanosheets, hollow and coralloid nanostructures via controlling reaction temperature, reaction time, precursors, etc^21^,^22^,^23^,^24^,^25^. Generally, metal ions with a reduction potential of −0.27 volts or higher present in MOFs form metal NPs during thermolysis in N_2_, whereas metal ions with a reduction potential lower than −0.27 volts form metal oxide NPs during thermolysis in N_2_. MIL-88A as an important kind of MOFs was synthesized by linking Fe(III) to the oxygen atoms of fumaric acid regularly^26^. The ordered structure effectively prevented the aggregation of Fe_3_O_4_ nanoparticles and the unsaturated organic linker not only acted as reducing agent but also could be further transformed into porous carbon when MIL-88A was decomposed to Fe_3_O_4_^27^. Recently, Hee Jung Lee *et al.* synthesized magnetic particle-embedded porous carbon composites from MIL-88A under relatively high temperature^28^. Differing from their work, the present work focused on the transformation process of MIL-88A when it was calcinated from 200 °C to 500 °C. Furthermore, the relationship between the structure of precursors and morphologies of products was also presented in this work. We found that, calcinated at low temperatures, the MIL-88A could convert to 3D hierarchical Fe_3_O_4_/carbon superstructures with controllable particle size and shape and performed good electrical conductivity due to the carbon matrix enhanced the electrochemical property of the nanocomposites (Fig. 1). Although remarkably significant progress has been obtained in shape-controlled synthesis of MOFs so far, MOF-derived Fe_3_O_4_@carbon with different particle sizes and morphologies have not been reported yet.

## Results and Discussion

**Figure 1:**
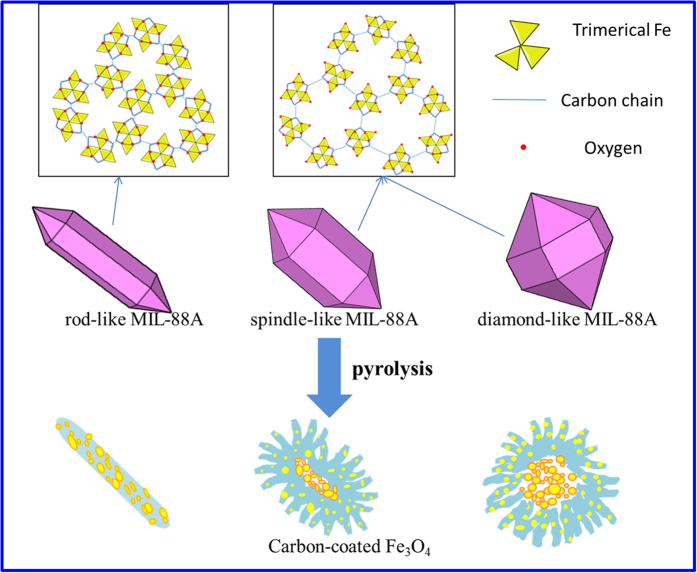
Schematic illustration of the formation process of Fe_3_O_4_@C nanostructures derived from MIL-88A.

**Figure 2:**
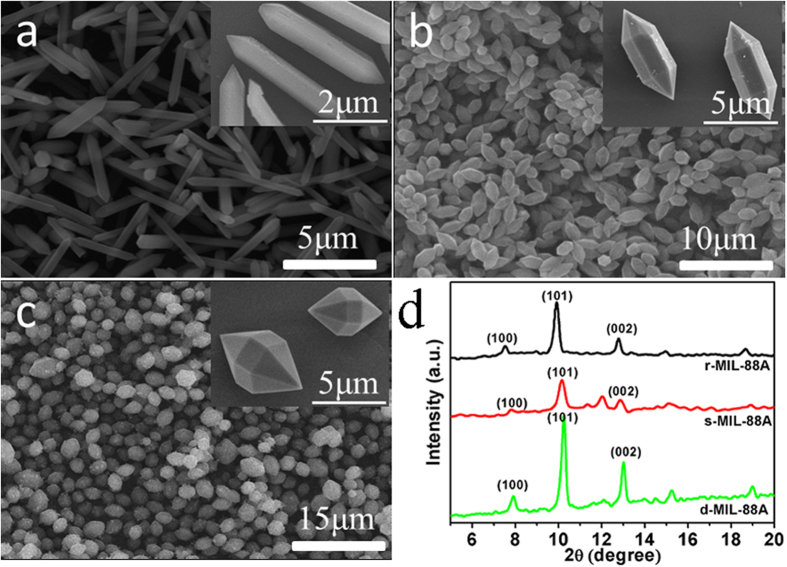
(**a–c**) SEM images of MIL-88A crystals with different structures: r-MIL-88A (**a**), s-MIL-88A (**b**) and d-MIL-88A **(c**). (**d)** XRD patterns of various MIL-88A: r-MIL-88A, s-MIL-88A and d-MIL-88A.

Porous carbon coated Fe_3_O_4_ was synthesized based on the solid-template method. The hierarchical Fe_3_O_4_/carbon superstructures with different morphologies can be achieved by pyrolysis of MIL-88A with different morphologies as depicted in Figure 1. The MIL-88A with different morphologies were successfully synthesized by changing the solvent and the concentration of FeCl_3_·6H_2_O. Fig. 2a–c showed scanning electron microscopy (SEM) images of MIL-88A crystals prepared under different conditions. The rod shaped small size particle with an average diameter of 500 nm was shown in Fig. 2a. The spindle-like particles with an average diameter of 1 μm and the diamond-shaped large size precursor with diameter of 5 μm were presented in Fig. 2b and c, respectively. The particle size is crucially determined by the nucleation rate. In general, fast nucleation gives a large number of nuclei, and shortens the crystal growth stage, leading to small-sized particles. In contrast, slow nucleation gives a smaller number of nuclei, and elongates the growth stage, leading to large-sized particles^29^. Considering the solvation effect, the stronger solvation of Fe^3+^ ions in N,N-dimethyl formamide (DMF, μ = 3.86D) solution drastically slowed down the generation speed of MIL-88A crystals, leading to large-sized MIL-88A crystals. In water (μ = 1.85D), the nucleation quickly proceeded to generate small-sized nanoparticles with high yield. For middle-sized MIL-88A, FeCl_3_·6H_2_O concentration was decreased to 2.4 mmol and two reactants of FeCl_3_·6H_2_O and fumaric acid were mixed beforehand. The first mixture made Fe^3 + ^reacted directly with fumaric acid as soon as DMF was added. Therefore, the middle-sized particle appeared due to mild crystallization speed. As comparison, the product synthesized by dissolving 2.4 mmol FeCl_3_·6H_2_O and fumaric acid in 10 ml ultra–pure water separately was also studied and the diameter was found to be 10 μm.

X-Ray powder diffraction (XRD) measurements (Fig. 2d) were performed to examine the crystal structure of the resulted three kinds of MIL-88A samples with different morphologies (r-MIL-88A, s-MIL-88A and d-MIL-88A). All these X-ray diffraction patterns of prepared samples were consistent with the well-known MIL-88A crystal structure^30^. The different shapes were determined by the growth rates along different directions. In this case, the (100), (101), (002) crystallographic facets developed apparently and other diffraction peaks at 2θ = 11°, 12°, 14.5° of s-MIL-88A and d-MIL-88 were stronger than that of r-MIL-88A. Compared with r-MIL-88A, the right shift was observed at the diffractions of (100), (101), (002) crystallographic facets of s-MIL-88A and d-MIL-88. The shift might be due to the solvent absorption and swelling effect.

For the present work, the MOF-template method was used to prepare Fe_3_O_4_-carbon hierarchical nanocomposites. The progress could be followed by thermo-gravimetric analysis (TGA) curve as shown in Fig. S1 (Supporting Information). The first main mass loss stage was due to the volatilization of the solvent (H_2_O or DMF) accompanied by slight degradation of fumaric acid. The further degradation from 200 °C to 300 °C was consistent with the breakdown of fumaric acid in a similar range (200–250 °C). Another degradation from 300 °C to 500 °C was observed and the XRD characterization indicated that the conversion from Fe_2_O_3_ to Fe_3_O_4_ occurred at this stage (discussed in the following). The conversion could be attributed to the incomplete calcined products as evidenced by thermal stability^27^. The phase corresponding to the Fe_3_O_4_@C nanocomposites was stabilized at 500 °C. After 500 °C, the MIL-88A was totally decomposed thus no more mass loss was observed. The carbon generated during the calcination which has been proven in XPS full-spectra of Fe_3_O_4_@C_400_ (Fig. S2, Supporting Information) could act as a buffer to prevent aggregation of metal oxides^24^.

The XRD patterns of FeO_x_@C_200,_ FeO_x_@C_300_ and FeO_x_@C_400_ (Here, X was used to represent the iron oxide due to the uncertain of the proportion of iron and oxygen) were also shown in Fig. S3 (Supporting Information). The diffraction peaks of FeO_x_@C_200_ at 2θ = 24°, 32°, 35°,41°, 49°, 54°, 62°, 64° in all XRD patterns matched well with crystal planes of pure solid α-Fe_2_O_3_ (Hematite, JCPDS card no. 06–0502, curve a). Both the characteristics diffraction peaks of Fe_2_O_3_ and Fe_3_O_4_ were simultaneously observed on the XRD patterns of FeO_x_@C_300_. During the thermal decomposition, the relatively high temperature induced the conversion of Fe_2_O_3_ into Fe_3_O_4._ The XRD patterns of FeO_X_@C_400_ as shown in pattern c, the characteristic diffraction peaks at 30°, 36°, 43°, 54°, 57°, and 63° were indexed as the diffractions of the (220), (311), (400), (422), (511), and (440) crystalline planes of Fe_3_O_4_ according to the standard spectrum of magnetite (JCPDS card no. 19–629).

**Figure 3:**
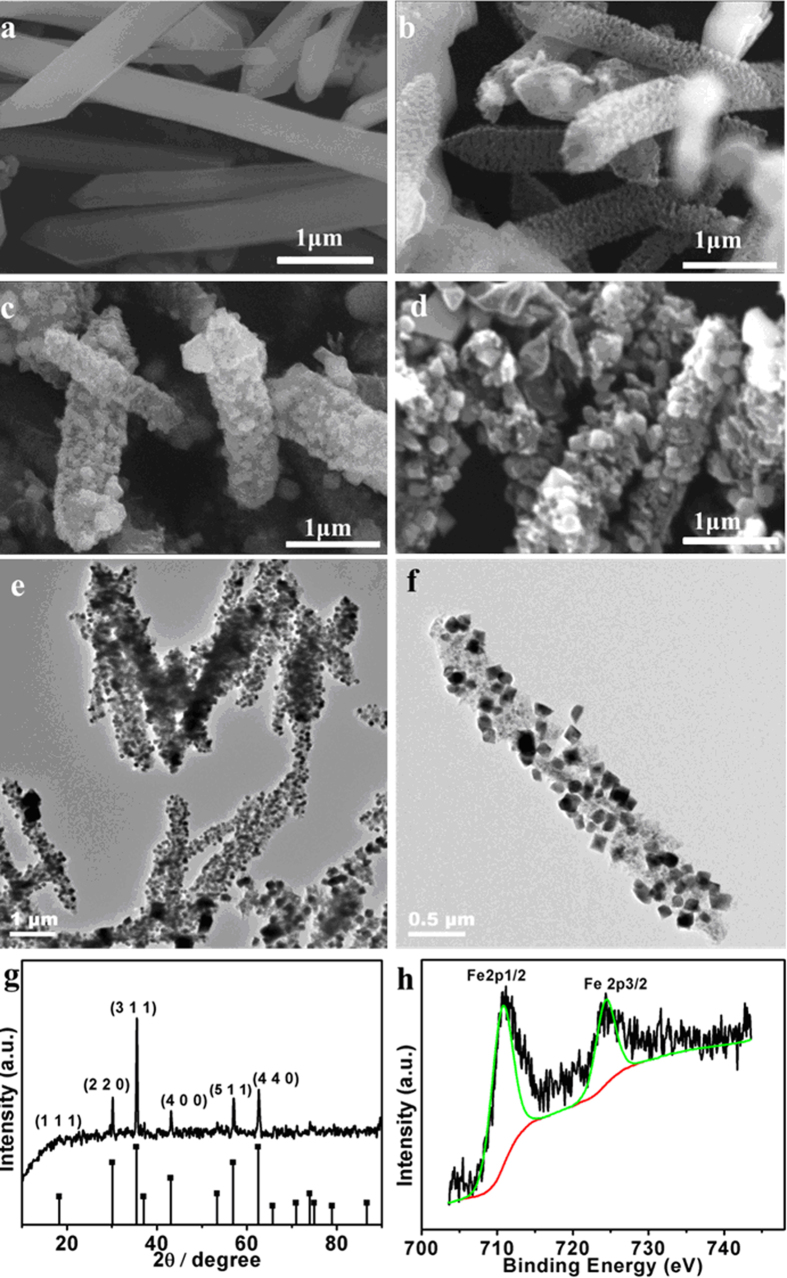
(**a–d**) SEM images of r-MIL-88A calcined at different temperature: 200 °C (**a**), 300 °C (**b**), 400  °C (**c**), 500 °C (**d**). (**e,f**) TEM images of Fe_3_O_4_@C_r_ by calcining r-MIL-88A at 400 °C. (**g**) XRD patterns of Fe_3_O_4_@C_r_ by calcining r-MIL-88A at 400 °C. (**h**) XPS of Fe 2p spectrum of Fe_3_O_4_@C_r_ derived from r-MIL-88A at 400 °C for 30 min.

SEM and transmission electron microscopy (TEM) were both employed to reveal the morphology change of the three kinds of samples at different pyrolysis temperatures at N_2_. In the first temperature gradient, MIL-88A was heated at 200 °C for 30 min, the rod-shape of r-MIL-88A was kept and the formation of Fe_2_O_3_ was indicated by XRD while the edge disappeared (Fig. 3a). In the second stage (300 °C), iron oxide was formed on the near-surface and there were spherical FeO_x_ particles decorating the incomplete calcined precursors (Fig. 3b). The long holding time induced the conversion of hematite into magnetite incompletely due to organic residues acting as reducing agents^27^. The further studies confirmed that the total heating time of over 40 min was required to complete such decomposition and the conversion of hematite into magnetite was evidenced by XRD pattern and X-ray photoelectron spectroscopy (XPS) spectra (discussed in the following). Growth of iron metal crystal was induced by increasing the calcined temperature to 400 °C (Fig. 3c). Further increasing the annealing temperature to 500 °C and holding this temperature for 30 min caused the enlarged iron oxide particles as a result of crystal aggregation (Fig. 3d). TEM images (Fig. 3e,f) for rod-shape materials calcined at 400 °C gave further evidence of the composite structure showing Fe_3_O_4_ coated with porous carbon. It could be established from Fig. 3e,f that the Fe_3_O_4_ nanoparticles with average diameter of 100 nm were encapsulated individually by a thin carbon boundary and such particles were dispersed in a porous carbon matrix. When the thermal treatment was performed at 400 °C for 30 min in N_2_ atmosphere with a heating rate of 5 °C/min, the products had shapes of parent precursors and improved Fe_3_O_4_ content. The XRD (Fig. 3g) revealed that the diffraction peaks of Fe_3_O_4_ became more intense and sharper in the FeO_x_@C_400_, again providing evidence of the growth of Fe_3_O_4_ crystallites and the structural evolution at elevated temperatures. The XPS spectroscopy was employed to identify the composition of the products synthesized at 400 °C. The binding energy values of 710.8 eV and 724.6 eV for Fe 2p3/2 and 2p1/2, respectively, as shown in XPS spectra (Fig. 3h) were close to the published Fe_3_O_4_^31^. The analysis data of XPS of Fe 2p3/2 spectra (Fig. S4A, Supporting Information) indicated the conversion rate from Fe_2_O_3_ to Fe_3_O_4_ was as high as 91.1 w% for r-MIL-88A.

**Figure 4:**
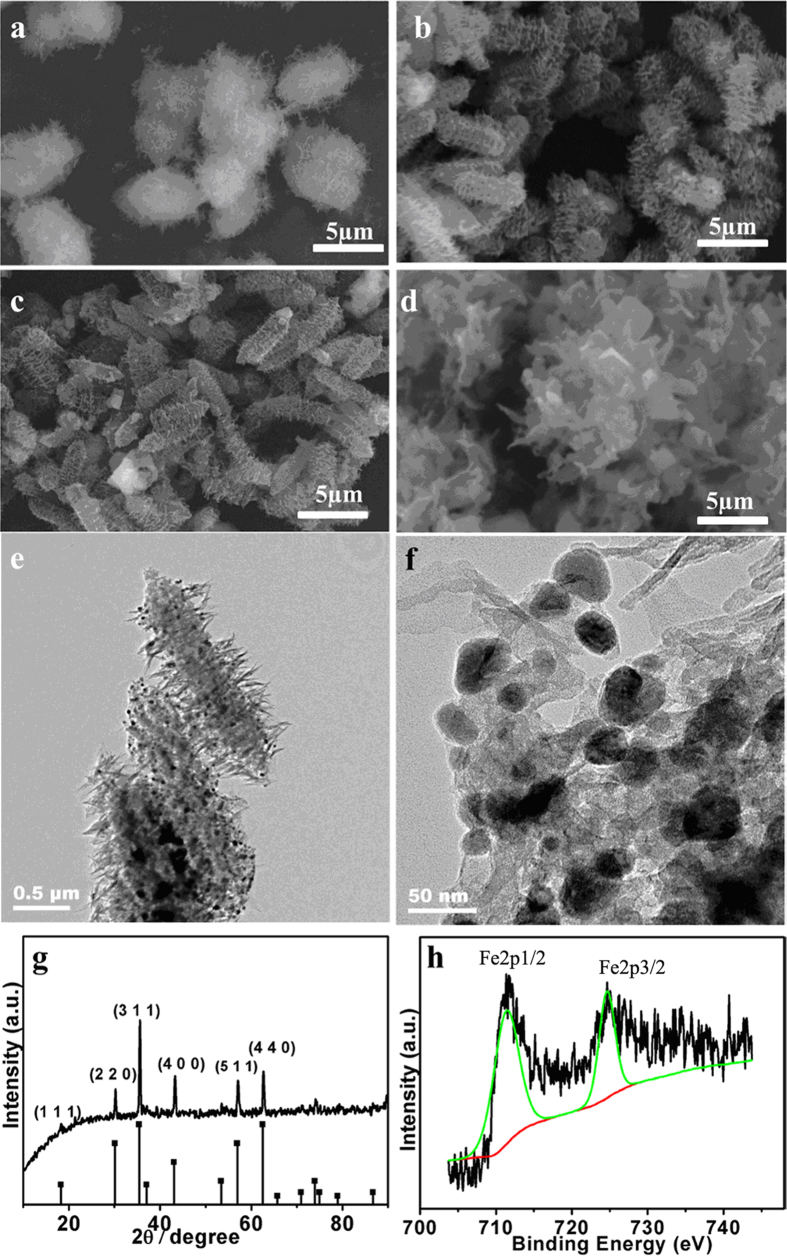
(**a–d**) SEM images of s-MIL-88A calcined at different temperature; 200 °C (**a**), 300 °C (**b**), 400 °C (**c**), 500 °C (**d**). (**e,f**) TEM images of Fe_3_O_4_@C_s_ by calcining s-MIL-88A at 400 °C. (**g**) XRD patterns of Fe_3_O_4_@C_s_ by calcining s-MIL-88A at 400 °C. (**h**) XPS of Fe 2p spectrum of Fe_3_O_4_@C_s_ derived from s-MIL-88A at 400 °C for 30 min.

For s-MIL-88A, the volatilization of solvent in the first stage led the smooth surface to be rough (Fig. 4a) and the burrs of product at 300 °C converted into bulk (Fig. 4b). No obvious change was found when the temperature was increased from 300 °C to 400 °C (Fig. 4c). The TGA curve of s-MIL-88A in this range was more moderate than that of r-MIL-88A (Fig. S1, Supporting Information). When the temperature surpassed 400 °C, the decomposition of the precursors was very quickly thus the iron oxide was aggregated significantly (Fig. 4d). As shown by the TEM image given in Fig. 4e,f, the size of Fe_3_O_4_ crystals was less than 50 nm and dispersed uniformly in dendritic carbon matrix. Different from other MO_x_@C (MO_x_: metal oxides) derived from MOFs, the s-MIL-88A transformed to dendritic shape rather than a smooth and compact surface, which could increase the specific surface area and utilization rate of the MO_x_. The XRD (Fig. 4g) and XPS (Fig. 4h) spectroscopy were employed to identify the composition of the products prepared at 400 °C. The characteristic diffraction peaks at 30°, 36°, 43°, 54°, 57°, and 63° were indexed as the diffractions of the (220), (311), (400), (422), (511) and (440) crystalline planes of Fe_3_O_4_ according to the standard spectrum of magnetite and no other crystalline planes was found in the XRD pattern. The binding energy values of 710.8 eV and 724.6 eV were ascribed to Fe 2p3/2 and 2p1/2, respectively (Fig. 4h). The analysis data of XPS of Fe 2p3/2 spectra^32^ (Fig. S4B, Supporting Information) indicated the conversion rate of Fe_2_O_3_ to Fe_3_O_4_ was as high as 81.5 w% for s-MIL-88A.

**Figure 5:**
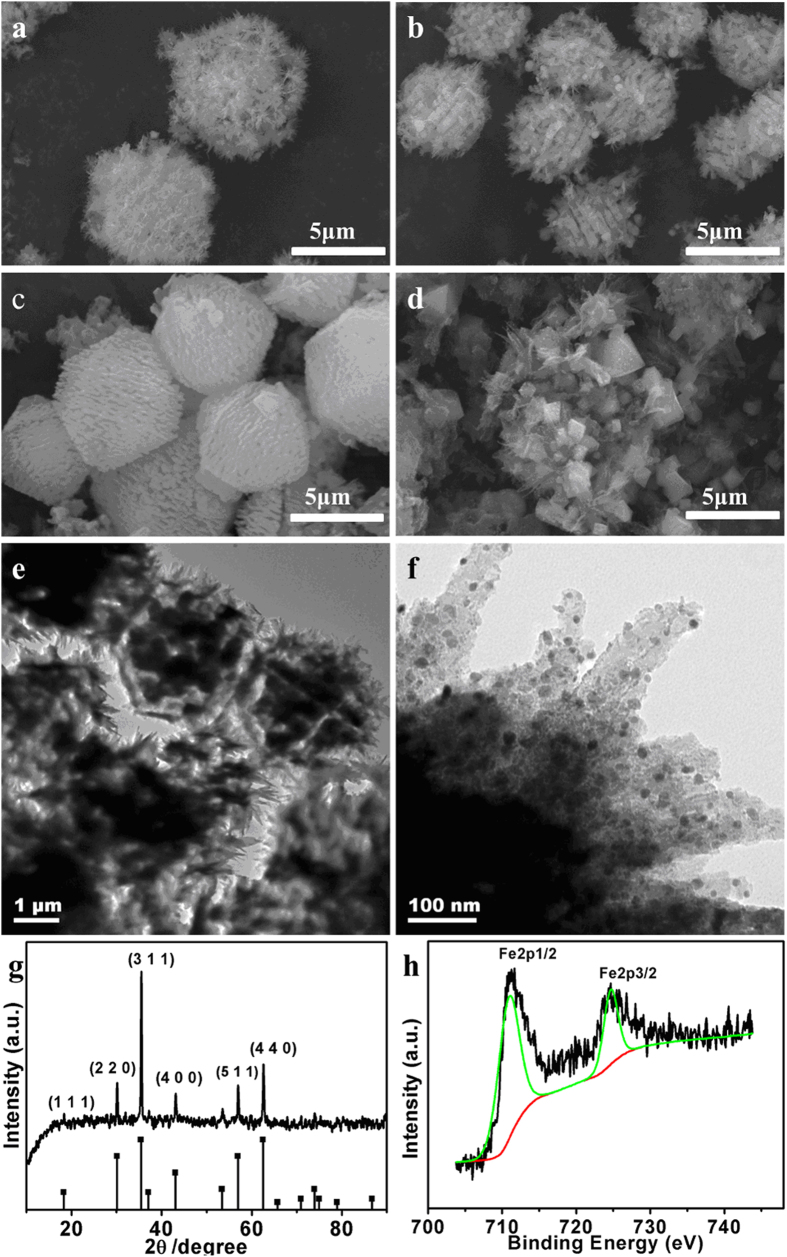
(**a–d**) SEM images of d-MIL-88A calcined at different temperature; 200 °C (**a**), 300 °C (**b**), 400 °C (**c**), 500 °C (**d**). (**e,f**) TEM images of Fe_3_O_4_@C_d_ by calcining d-MIL-88A at 400 °C. (**g**) XRD patterns of Fe_3_O_4_@C_d_ by calcining d-MIL-88A at 400 °C. (**h**) XPS of Fe 2p spectrum of Fe_3_O_4_@C_d_ derived from d-MIL-88A at 400 °C for 30 min.

The situation for d-MIL-88A was similar to the above one. The diamond-like materials obtained at different temperatures with distinct morphologies and structures were shown in Fig. 5. The bulk crystal was formed at 500 °C (Fig. 5d). At relatively low temperature of 200–400 °C, the morphology of the products was similar to dandelion and retained the size of d-MIL-88A precursor particles (Fig. 5a–c). The TEM images (Fig. 5e,f) of the nanocomposites prepared at 400 °C established a ball-in-dendritic carbon shell structure. The Fe_3_O_4_ crystals were dispersed in the dendritic carbon shell with size of about 20 nm. As shown in the SEM (Figs 3, 4 and 5), the surface of r-MIL-88A at 200 °C was compact while the s-MIL-88A and d-MIL-88A at 200 °C were fluffy. The difference was first related to the absorption of solvent. The cell parameter is a direct measurement of distance between Fe and trimeric units and the amplitude of the swelling is influenced by the absorption of polar solvent. Compared with H_2_O, the stronger polar moment of DMF could make the MIL-88A present bigger cell parameter which indicated the longer distance between the inorganic trimeric units and larger swelling amplitude of s-MIL-88A and d-MIL-88A^33^. The heating would lead to solvent volatilization while the topology of the framework was maintained thus the volatilization of DMF resulted in larger voids than H_2_O. The voids and burrs formed when s-MIL-88A and d-MIL-88A heated at 400 °C revealing by the TEM further proved the inference. However, the voids and burrs weren’t observed during the pyrolysis process of r-MIL-88A due to the smaller cell parameter. On the other hand, the long distance between Fe and trimeric units of s-MIL-88A or d-MIL-88A played as a buffer for the aggregation of Fe_3_O_4_ crystal thus led to small particle size. In addition to the volatilization of solvent, the decomposition of organic ligand also attributed to the porosity of the hierarchical nanostructure. According to previous works^24^,^34^, we could deduce that the amorphous carbon generated from the decomposition of organic ligands of MIL-88A served as a temporary framework to distribute FeOx particles. As the temperature increased, the MIL-88A contracted inward and the organic framework further decomposed into carbon and gas (CO_2_ and hydrocarbons) under N_2_ atmosphere. The adhesive force owing to the volume loss and the release of internally generated gases prevented the inward contraction of Fe_3_O_4_-carbon shell. Finally, for d-MIL-88A, the hierarchical nanocomposites with compact Fe_3_O_4_-carbon core and loose shell were formed. While for s-MIL-88A, the smaller diameter didn’t allow the formation of apparent voids between the core and dendritic carbon shell.

The intensive and sharp diffraction XRD peaks (Fig. 5g) revealed the growth of Fe_3_O_4_ crystallites and the structural evolution at elevated temperatures. The XPS spectroscopy of the products from d-MIL-88A synthesized at 400 °C was similar to that from s-MIL-88A at 400 °C (Fig. 5h and Fig. S4C in Supporting Information). The XPS indicated the conversion rate of Fe_2_O_3_ to Fe_3_O_4_ was 77.5% for d-MIL-88A.

Nitrogen adsorption–desorption isotherms shown in (Fig. S5A Supporting Information) were measured to evaluate the specific surface area and the pore size distribution of Fe_3_O_4_@C_400_. The curve for Fe_3_O_4_@C_400_ samples was a little bit similar to the I-type isotherm and suggested the different pore sizes spanning from micro to macropores. The steep increase at low relative pressure pointed the existence of micropores. Hysteresis between adsorption and desorption branches could be observed at medium relative pressure for r-MIL-88A and s-MIL-88A, which demonstrated the existence of mesopores. The steep increase at the tail of the relative pressure near to 1.0 revealed the presence of macroporosity. The majority of the pores were located in the region of mesopore. All the samples displayed very close pore size distribution with a peak centering at ca. 3.0 nm as shown in the pore size distributions curve calculated from the nitrogen adsorption branches (Fig. S5B, Supporting Information). The specific surface area were calculated to be 70.3 cm^2^ g^−1^, 33.4 cm^2^ g^−1^ and 20.5 cm^2^ g^−1^ for r-Fe_3_O_4_@C_400,_ s-Fe_3_O_4_@C_400_ and d-Fe_3_O_4_@C_400_, respectively. The specific surface area was higher than many reported metal oxides. We deduce that the high specific surface area of r-Fe_3_O_4_@C_400_ might result from the small particle size of Fe_3_O_4_ which was estimated to be about 20–30 nm. Although there were voids between the porous shell and the core in d-Fe_3_O_4_@C_400,_ the compact core would lead to low specific surface area. Therefore, both the particle size and the structure should be both taken into consideration when synthesizing nanostructure with high specific surface area.

As an important member of transition-metal oxide family, Fe_3_O_4_ has been used as electrocatalytic material. Yan and co-workers have recently discovered that Fe_3_O_4_ magnetic nanoparticles (MNPs) actually exhibited an intrinsic peroxidase-like activity^35^. A significant amount of research has been focused on imitating peroxidase activity with various noble metals (e.g., Au, Pt and Pd) modified Fe_3_O_4_ MNPs^36^,^37^,^38^. The Fe_3_O_4_@C for amino acid sensor has also been reported^39^. In view of the good electrochemical property, the hierarchical Fe_3_O_4_/carbon superstructures prepared here were employed to sensing N-acetyl cysteine.

**Figure 6:**
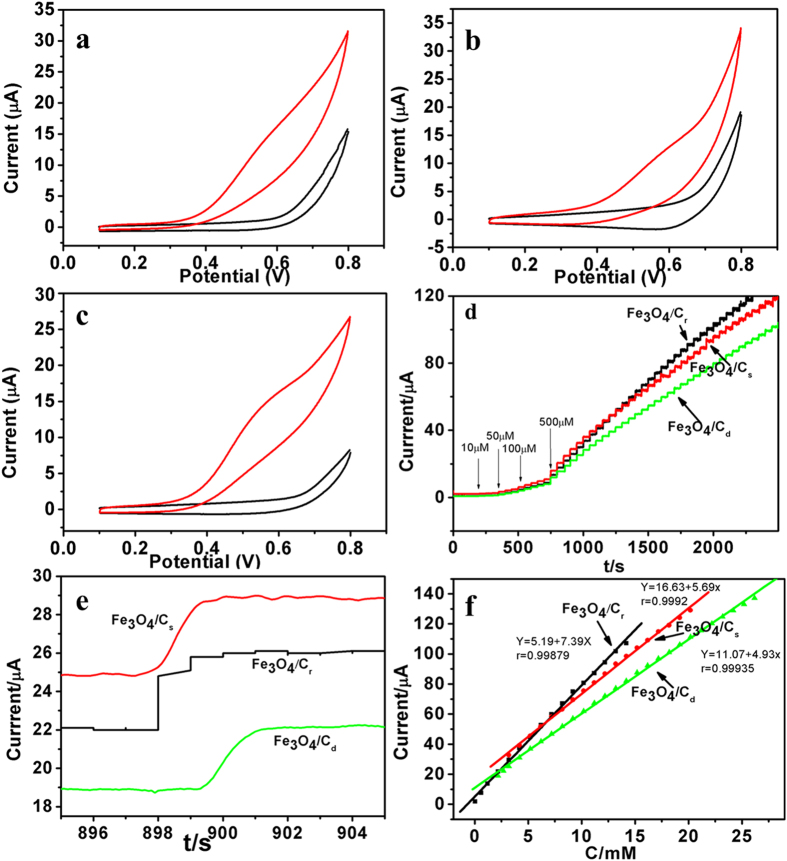
(**a–c**) CVs of different electrodes in 0.1 M NaOH in the absence and presence of 0.3 mM N-acetyl cysteine. Scan rate: 50 mV s^−1^ ((**a**) Fe_3_O_4_@C_r_, (**b**) Fe_3_O_4_@C_s_, and (**c**) Fe_3_O_4_@C_d_). (**d**) Typical amperometric responses of Fe_3_O_4_@C/GCE to successive injection of N-acetyl cysteine into the stirred 0.1 M NaOH. (**e**) A segment of the amperometric concentration step response showing sensor response time; (**f**) The calibration curve of amperometric responses ((**a**) Fe_2_O_3_@C_r_, (**b**) Fe_3_O_4_@C_s_, and (**c**) Fe_3_O_4_@C_d_).

Cyclic voltammograms (CVs) of different modified electrodes (Fe_3_O_4_@C_r_/GCE (glassy carbon electrode), Fe_3_O_4_@C_s_/GCE, Fe_3_O_4_@C_d_/GCE and bare GCE) in 0.1 M NaOH in the presence of N-acetyl cysteine were shown in Fig. 6a–c and Figure S6 When 0.3 mM N-acetyl cysteine was added into 0.1 M NaOH, the catalytic current obtained on Fe_3_O_4_@C modified electrodes increased obviously and were much larger than that obtained on bare GCE, indicating that Fe_3_O_4_@C had good catalytic activity for N-acetyl cysteine. The CVs and amperometry were carried out to explore how the morphology affected the electrochemical performance of the three kinds of Fe_3_O_4_@C/GCE. The CVs of Fe_3_O_4_@C/GCE at varied scan rate was investigated in 0.1 M NaOH. As shown in Figure S7A-C (Supporting Information), the anodic peak current density increased as the scan rates increased from 10 to 400 mV s^−1^. The peak current was proportional to the square root of scan rates as shown in the inset of Figure S7A–C (Supporting Information), indicating this process for the three kinds of Fe_3_O_4_@C_r_/GCE were all diffusion-controlled. Furthermore, the oxidation of N-acetyl cysteine at Fe_3_O_4_@C were started at about 300 mV then increased sharply towards the positive potential. A weak peak centered at about 600 mV which was chosen as the working potential in the following experiments.

Amperometric measurements were carried out at 0.6 V by successive injection of N-acetyl cysteine (Fig. 6d) into a stirring 0.1 M NaOH on Fe_3_O_4_@C_r_/GCE, Fe_3_O_4_@C_s_/GCE and Fe_3_O_4_@C_d_/GCE, respectively. The oxidation current density reached a maximum steady-state value within 2 s (Fig. 6e). Each current response presented a linear dependence upon the concentration of N-acetyl cysteine as shown in Fig. 6f. Fe_3_O_4_@C_r_/GCE showed a linearity (*I*(μA) = 5.19 + 7.39 *C*, *R* = 0.99879, line a) in the range of 0.007–14.18 mM. The detection limit was estimated to be 2 μM based on the criterion of a signal–to–noise ratio of 3 (R/N = 3). The linearity got from Fe_3_O_4_@C_s_/GCE (*I*(μA) = 16.63 + 5.9 *C*, *R* = 0.9992, line b) showed a linear range of 0.028–20.2 mM and a detection limit of 8 μM. Fe_3_O_4_@C_d_/GCE showed a linearity (*I*(μA) = 5.19 + 7.39 *C*, *R* = 0.99879, line c) in the range of 0.086–26.2 mM and a detection limit of 26 μM. A comparison of the assay performance of Fe_3_O_4_@C/GCE sensor prepared here with other Fe_3_O_4_-based sensors were shown in Table S1 (Supporting Information). It could be clearly seen that the sensor based on the novel hierarchical Fe_3_O_4_/carbon superstructures possessed better analytical performances.

Fe_3_O_4_@C_r_ with the smaller particles had lower detection limit. Figure 6e was a segment of amperometric response of Fe_3_O_4_@C/GCE in 0.1 M NaOH in the presence of N-acetyl cysteine. It exhibited that the required time for Fe_3_O_4_@Cr to achieve stable current was shorter than the other two electrodes. Since the electrocatalytic process was diffusion-controlled, it might be deduced that the smaller dimension provided more space and sites to contact with N-acetyl cysteine thus it would have better catalytic ability. However, Fe_3_O_4_@C_s_/GCE and Fe_3_O_4_@C_d_/GCE had a wider detection range. It could be attributed to the specific shape. As the reaction proceeded, more and more by-products would absorb on the surface of Fe_3_O_4_@C particle, which reduced the catalytic activity gradually. For dendritic-liked Fe_3_O_4_@C_s_ and Fe_3_O_4_@C_d_, N-acetyl cysteine could diffuse into their inner and be catalytically oxidized by the inner surface. The loading amount of Fe_3_O_4_ in the r-Fe_3_O_4_@C_400_, s-Fe_3_O_4_@C_400_ or d-Fe_3_O_4_@C_400_ nanocomposites could be estimated by TGA curve as shown in Fig. S8 (Supporting Information). It obviously showed that the loading amount of Fe_3_O_4_ of r-Fe_3_O_4_@C_400_, s-Fe_3_O_4_@C_400_ and d-Fe_3_O_4_@C_400_ was about 82%, 67% and 62%, respectively. It can be easily concluded that the more the loading amount of Fe_3_O_4_, the better the catalytic property of the nanocomposite, as shown in Fig. 6f.

Interference is inevitable in the determination of some analyses. So, we have investigated the selectivity of the modified electrode in this work towards several possibly coexisted substances. Fig. S9 (Supporting Information) showed the current responses of the modified electrode toward some chemicals, including BrO_3_^–^, IO_3_^–^, NO_2_^–^, Cl^–^, NO_3_^–^, SO_4_^2-^, K^+^, Na^+^ and Mg^2+^. We presumed there was no interference if the variance of the catalytic current was smaller than 6% after the injection of other chemicals. It was obvious that chemicals such as saturated BrO_3_^–^ and IO_3_^–^, NO_2_^–^, Cl^–^, NO_3_^–^, SO_4_^2–^, K^+^, Na^+^ and Mg^2+^ in a 10-fold of N-acetyl cysteine concentration did not show obvious interference to 1 mM N-acetyl cysteine detection. The result implied the good selectivity of Fe_3_O_4_@C_s_/GCE. Similar results were also obtained for both Fe_3_O_4_@C_r_/GCE and Fe_3_O_4_@C_d_/GCE.

Chronoamperometry was employed to study the mass transfer kinetics and obtained the heterogeneous catalytic rate constant. Fig. S10A-C (Supporting Information) showed chronoamperograms recording with Fe_3_O_4_@C/GCE in the absence and presence (0.5 mM, 1.0 mM, 2.0 mM, 4.0 mM, 6.0 mM, 8.0 mM, 10.0 mM) of N-acetyl cysteine. The applied potential steps were set to 0.60 V and 0.30 V, respectively. Plotting the net current with respect to the minus square roots of time presented linear dependency (inset of Figure S10A-C). Therefore, diffusion-controlled process in the bulk solution was dominated for the oxidation of N-acetyl cysteine. Using the slope of the line, the diffusion coefficient of N-acetyl cysteine could be obtained according to Cottrell’s equation:


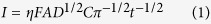


The catalytic rate constant (*K*_cat_) was calculated based on the slope of the *I*_cat_*/I*_d_ versus *t*^1/2^ plot as shown in the inset of Figure S10A-C according to the following equation:


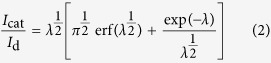


where *I*_cat_ and *I*_d_ was the current in the presence and absence of N-acetyl cysteine, respectively, *λ* = *K*_cat_*Ct* was the argument of the error function, *K*_cat_ was the catalytic rate constant and *t* was the consumed time. In the case where *λ* > 1.5, erf (*λ*^1/2^) was almost equal to unity, the above equation could be reduced to:


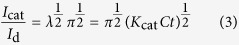


The mean value of N-acetyl cysteine diffusion coefficient and the catalytic rate constant (*K*_cat_) were listed in Table S2 (Supporting Information). These results further confirmed our conclusion that the material with smaller particle size and higher surface area showed better catalytic performance, meanwhile the dendritic shape could promote the diffusion of the electroactive material.

In summary we realized the transformation of Fe-containing MOF, a kind of typical porous material, into Fe_3_O_4_@C with different particle sizes. The different morphologies were determined by the cell parameter of precursors which was depended on the synthesis method since large cell parameter resulted in dendritic carbon and little Fe_3_O_4_ particles. The derived composites exhibited good conductivity and high electric catalytic activity due to the characteristics of the precursors such as porosity, tunability, regularity of structure, etc. What’s more, the results of electrochemisty experiments testified that the performances of MOF-derived materials were closely related to their morphology. Although the present study was focused on the discussion of particle sizes and shape, other factors, for example the size of pores, secondary building units, etc. could also be considered to optimize the desired materials. Finally, excepting serve as catalyst, recent progress on industrial level upscaling of MOF synthesis allows us to envision that such MOF-derived functional materials might play an important role in several application sectors in the future. For instance rechargeable batteries, supercapacitors, fuel cells and corrosion inhibition, MOF-derived materials are gaining momentum in the field of electrochemistry.

## Methods

### Materials

Fumaric acid and FeCl_3_·6H_2_O (99%) were obtained from Aladdin Industrial Corporation (Shanghai, China). NaOH (96%), N-acetyl cysteine and other chemicals were purchased from Beijing Chemical Reagent Factory (Beijing, China). All reagents were of analytical grade and used as received. All solutions were prepared with ultra–pure water, purified by a Millipore–Q system (18.2 MΩ cm).

### Instrumentation

Scanning electron microscopy (SEM) analysis was taken using a XL30 ESEM–FEG SEM at an accelerating voltage of 20 kV equipped with a Phoenix energy dispersive X-ray analyzer (EDXA). Transmission electron microscopy (TEM) analysis was taken using a JEM-2010(HR). X–ray powder diffraction (XRD) data were collected on a D/Max 2500 V/PC X–ray powder diffractometer using CuKα radiation (*λ* = 1.54056 Å, 40 kV, 200 mA). Thermogravimetric analysis (TGA) was conducted under a N_2_ flow with a heating rate of 5 °C/min, using an SDT 2960 instrument. Nitrogen adsorption-desorption isotherms were measured at −196 °C using a BELSORP-mini II instrument. Before the experiments, the samples were outgassed under vacuum at 40 °C. X-ray photoelectron spectroscopy analysis was taken using an AXIS ULTRA DLD at an accelerating voltage of 15 kV to study the element. All electrochemical measurements were performed on a CHI 660C electrochemical workstation (Shanghai, China) at ambient temperature. A conventional three–electrode system was employed including a bare or modified GCE as the working electrode, a platinum wire as the auxiliary electrode and a saturated calomel electrode (SCE, saturated KCl) as the reference electrode. The cyclic voltammetric experiments were performed in a quiescent solution. The chronoamperometry experiments were carried out under a continuous stirring using a magnetic stirrer. 0.1 M NaOH was used as the supporting electrolyte solution.

### Preparation of MIL-88A

For the synthesis of nano-sized MIL-88A crystals with different morphology, 4 mmol FeCl_3_·6H_2_O and 4.0 mmol fumaric acid were dissolved in 10 ml ultra–pure water separately. These two solutions were then mixed in equal volume and the mixture was transferred into a teflon reaction kettle, placed in an autoclave, and heated to 100 °C for 4 h. The as-synthesized MIL-88A rods were signed as r-MIL-88A. In order to synthesize MIL-88A with different morphology, the amount of iron source and the solvent were changed. 4 mmol FeCl_3_·6H_2_O and 4.0 mmol of fumaric acid were dissolved in 10 ml DMF separately, then the two solutions were mixed in a teflon reaction kettle. The diamond-shaped MIL-88A (hereafter abbreviated as d-MIL-88A) with a average size of 5 μm were successfully obtained after heating for 4 h at 100 °C. For spindle-like MIL-88A, 4.0 mmol fumaric acid was dissolved in 20 mL DMF and added into 2.4 mmol FeCl_3_·6H_2_O. The mixture was heated for 12 h at 100 °C in a teflon reaction kettle to form spindle-like MIL-88A (hereafter abbreviated as s-MIL-88A). Finally, the raw product was washed by DMF and deionized water for several times, respectively, and dried at 40 °C.

### Preparation of Hierarchical Fe_3_O_4_/Carbon Superstructures

The r-/s-/d-MIL-88A were placed in ceramic boats, transferred into a horizontal quartz tube and calcined in the horizontal tube furnace. The thermal treatment was performed at 400 °C for 30 min under N_2_ atmosphere with a heating rate of 5 °C/min from room temperature to 400 °C. Then the calcination was followed by natural cooling to room temperature under N_2_ atmosphere (the corresponding products were denoted as Fe_3_O_4_@C_r,_ Fe_3_O_4_@C_s_, Fe_3_O_4_@C_d_, respectively). To study the process of carbonization, the similar experiments were carried out at different target temperature to obtain FeO_x_@C_200,_ FeO_x_@C_300,_ FeO_x_@C_400_ and FeO_x_@C_500_.

### Preparation of Hierarchical Fe_3_O_4_/Carbon Superstructures Modified Electrode

Glassy carbon electrodes (GCEs, Φ = 2 mm) were carefully polished by 1.0, 0.3 and 0.05 μm Al_2_O_3_ powder in order. Then, the polished GCEs were scanned in 0.1 M KCl + 5.0 mM K_3_Fe(CN)_3_. After the same CVs with same peak current and same potential separation were obtained, the polished GCEs were used for the following experiments. The effective surface area of the GCEs was estimated to be about 0.0763 cm^2^. Simultaneously, 3.0 mg hierarchical Fe_3_O_4_/carbon superstructures were dispersed into 1.0 mL ultra–pure water to give 3.0 mg/mL homogeneous dispersions followed by adding 10 μL Nafion. Finally 10.0 μL suspensions were dropped on the polished GCE surface and dried in air.

## Additional Information

**How to cite this article**: Wang, L. *et al.* The MIL-88A-Derived Fe_3_O_4_-Carbon Hierarchical Nanocomposites for Electrochemical Sensing. *Sci. Rep.* **5**, 14341; doi: 10.1038/srep14341 (2015).

## Supplementary Material

Supplementary Information
