# Supplementary material for: The MIL-88A-Derived Fe3O4-Carbon Hierarchical Nanocomposites for Electrochemical Sensing
Source: Sci Rep. 2015 Sep 21;5:14341. doi: 10.1038/srep14341 (PMC4585678; doi:10.1038/srep14341)
Supplement: Supplementary Information [file srep14341-s1.doc]

Supplementary materials

**The MIL-88A-Derived Fe_3_O_4_-Carbon** **Hierarchical Nanocomposites for Electrochemical Sensing**

Li Wang*, Yayun Zhang, Xia Li, Yingzhen Xie, Juan He, Jie Yu and Yonghai Song*[[1]](#footnote-2)**

*College of Chemistry and Chemical Engineering, Jiangxi Normal University, 99 Ziyang Road, Nanchang 330022, China.*


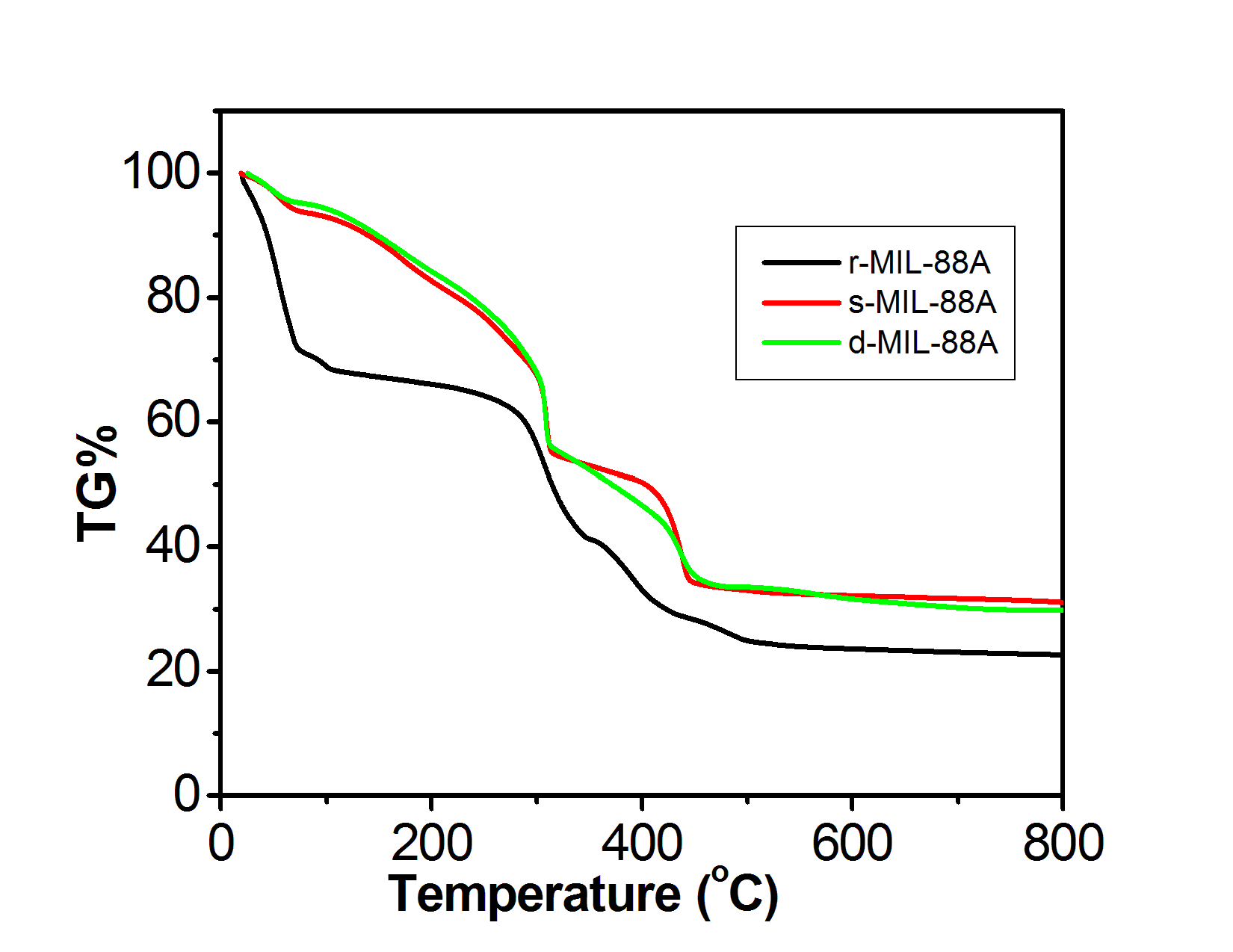


**Figure S1.** TGA of as-prepared MIL-88A at N_2_ atmosphere.


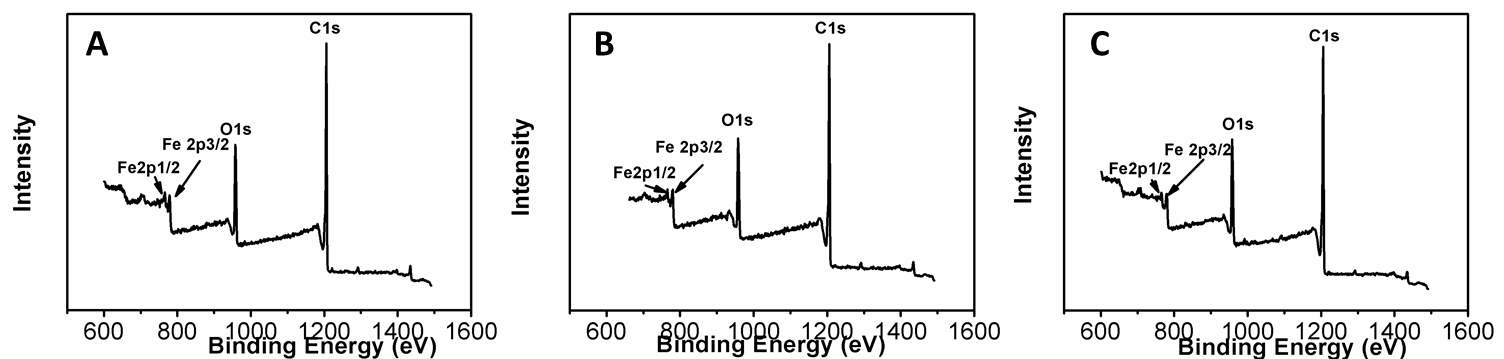


**Figure S2.** XPS full-spectra of Fe_3_O_4_@C_400_: (A) Fe_3_O_4_@C_r_, (B) Fe_3_O_4_@C_s_ and (C) Fe_3_O_4_@C_d_.

**Figure S3.** XRD patterns of metal oxide-doped carbons obtained by direct carbonization of MIL-88A crystals: (a) α-Fe_2_O_3_@C_200_, (b) FeO_X_@C_300_, and (c) Fe_3_O_4_@C_400_.


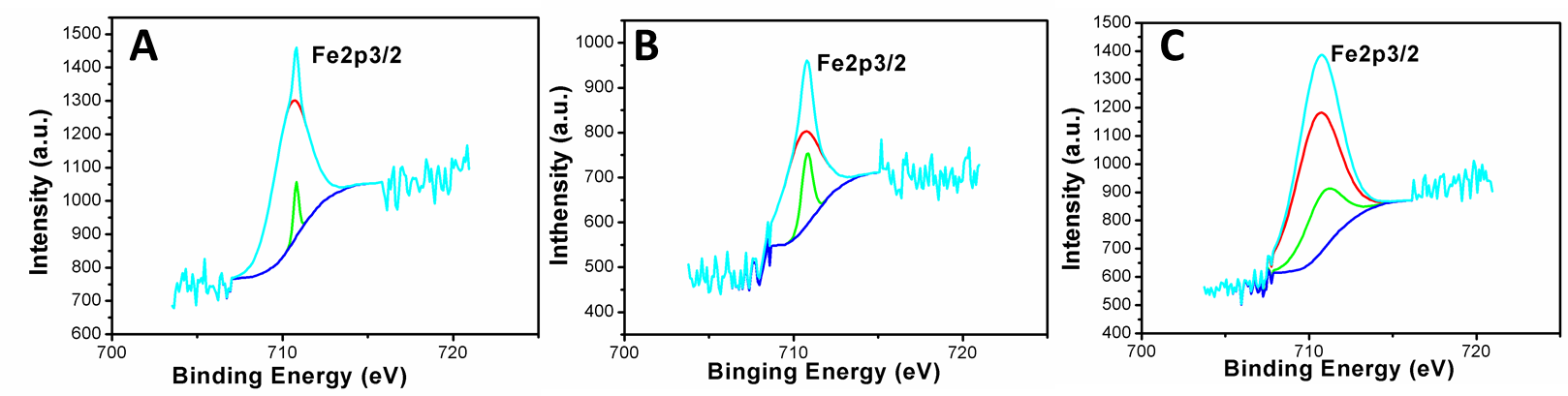


**Figure S4.** XPS of Fe 2p3/2 spectra of Fe_3_O_4_@C: (A) Fe_3_O_4_@C_r_, (B) Fe_3_O_4_@C_s_ and (C) Fe_3_O_4_@C_d_.


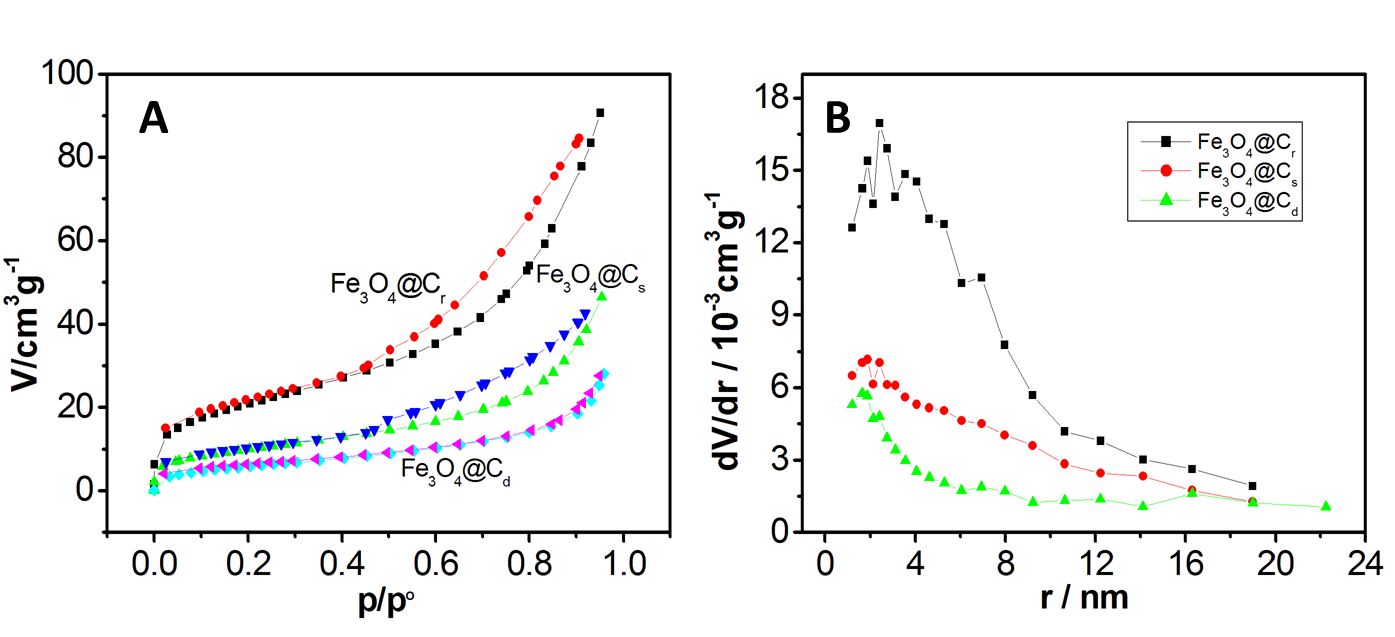


**Figure S5.** (A) Nitrogen adsorption–desorption isotherms and (B) pore size distributions calculated from N_2_ adsorption isothermals for Fe_3_O_4_@C samples.


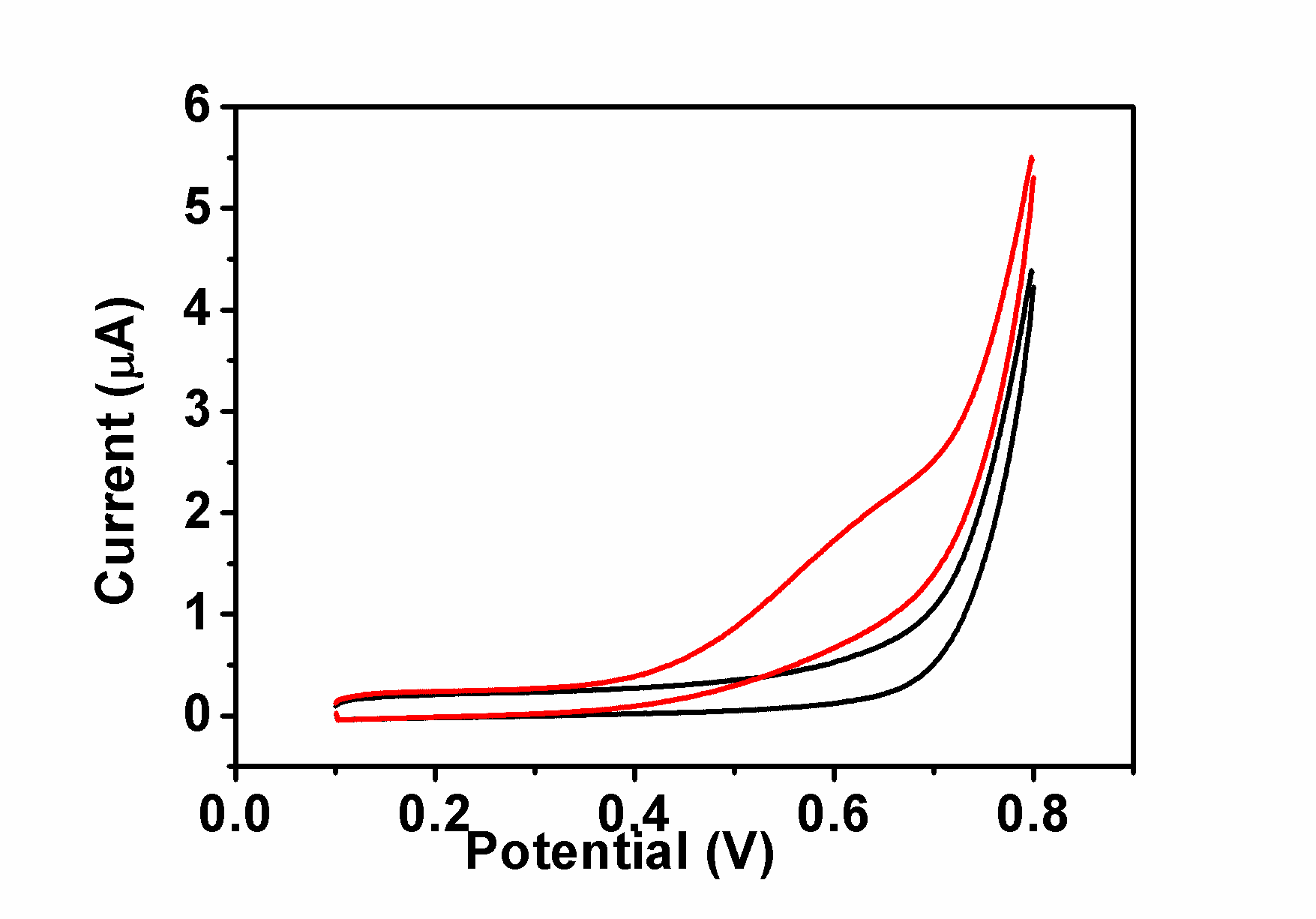

**Figure S6.** CVs of bare GCE in 0.1 M NaOH in the absence (black line) and presence (red line) of 0.3 mM N-acetyl cysteine. Scan rate: 50 mV s^−1^.

**
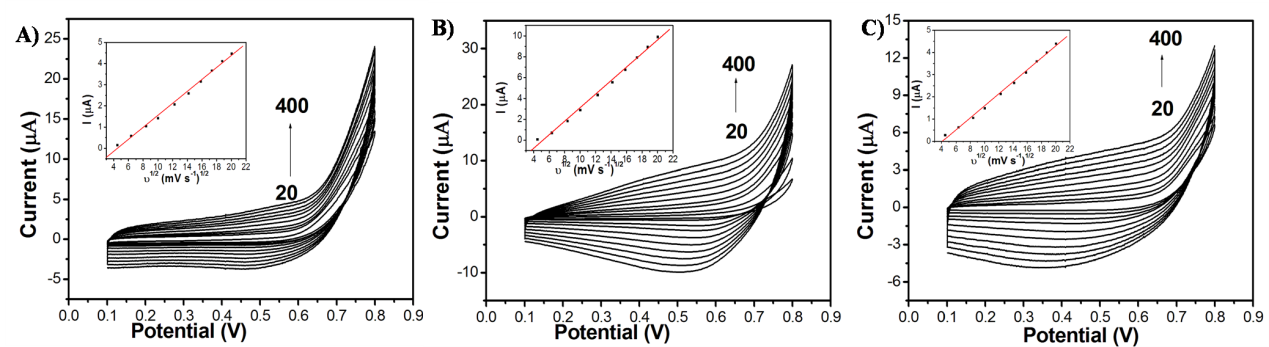
**

**Figure S7.** CVs of Fe_3_O_4_@C/GCE in 0.1 M NaOH at different scan rates (from inside to outside: 20, 40, 70, 100,150, 200, 250, 300, 350 and 400 mV s^−1^: (A) Fe_3_O_4_@C_r_/GCE, (B) Fe_3_O_4_@C_s_/GCE and (C) Fe_3_O_4_@C_d_/GCE). Inset: the plot of oxidation peak current at 500 mV versus the square root of the scan rate.


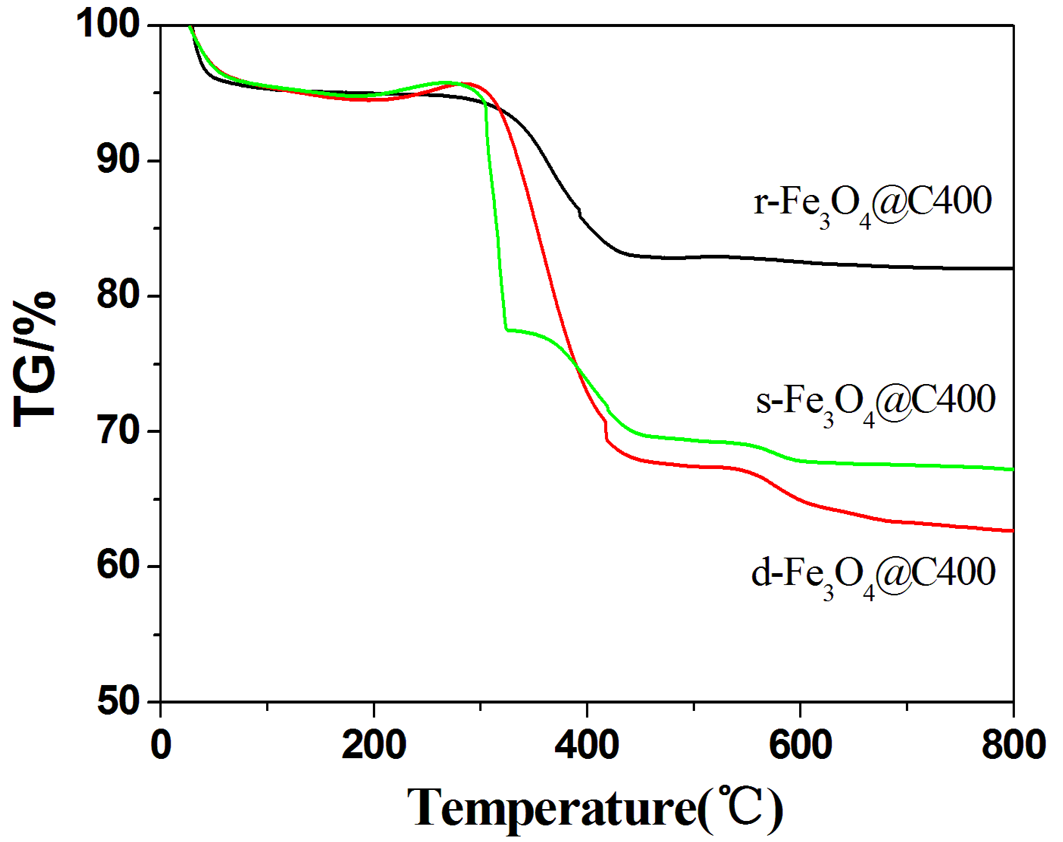


**Figure S8.** TGA of as-prepared Fe_3_O_4_@C_400_ at air.


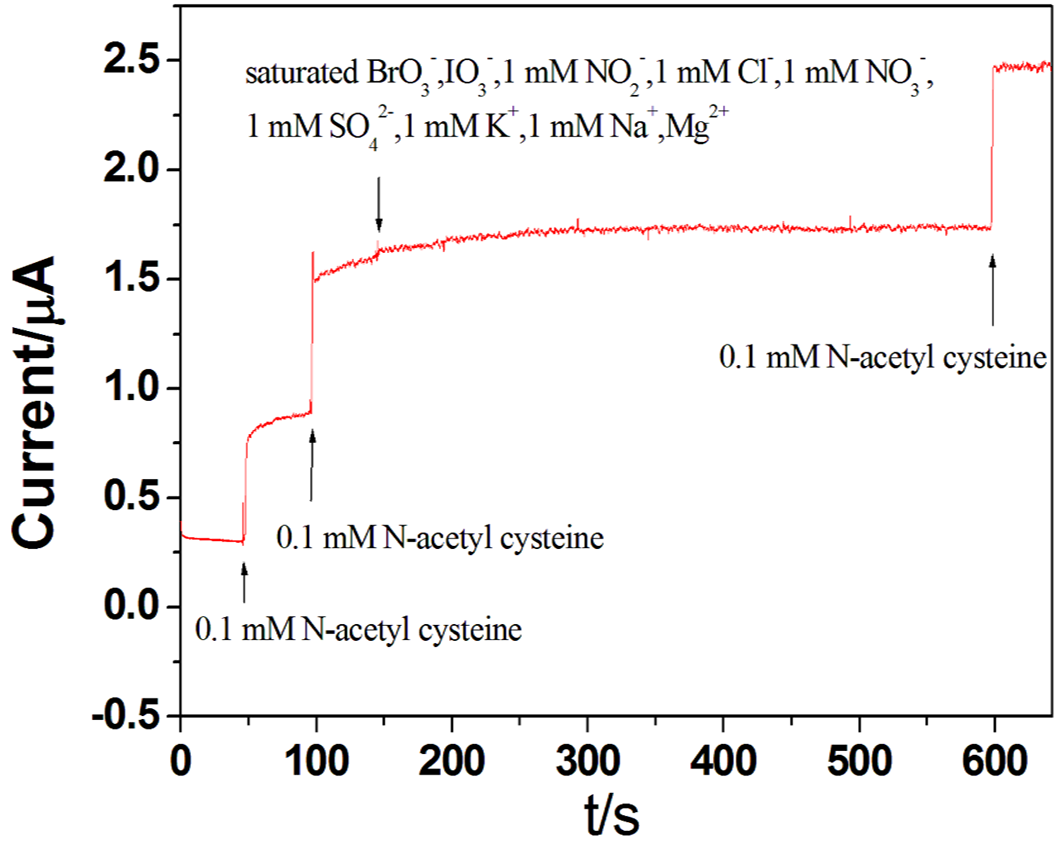


**Figure S9.** The interference effect of some possible coexisted substances on N-acetyl cysteine detection. Applied potential: 600 mV.

**
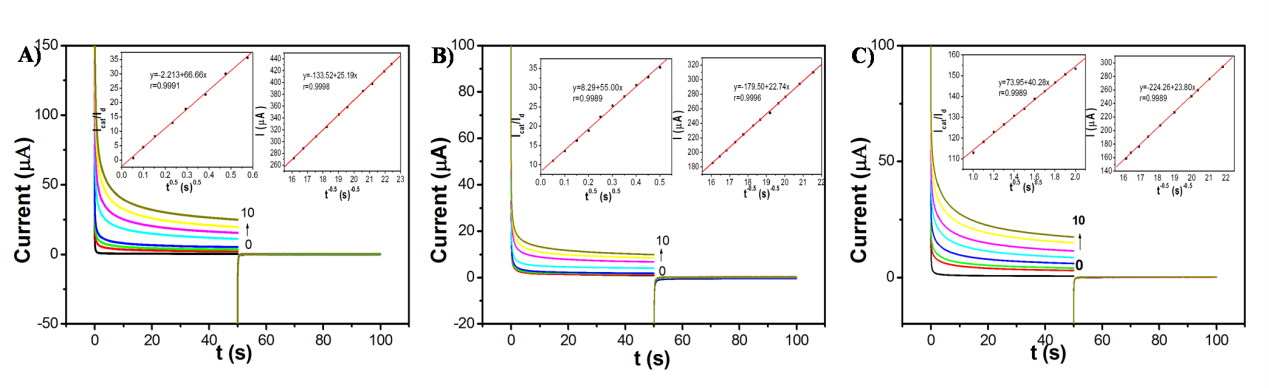
**

**Figure S10.** Chronoamperograms of Fe_3_O_4_@C/GCE in 0.1 M NaOH solution containing 0, 0.5, 1, 2, 4, 6, 8, 10 mM glucose (from bottom to up): (A) Fe_3_O_4_@C_r_/GCE, (B) Fe_3_O_4_@C_s_/GCE and (C) Fe_3_O_4_@C_d_/GCE. Potential steps were 600 mV and 300 mV, respectively.

**Table S1.** Comparison of the performance of various amino acid sensors.

| Modified electrode | Detection limit μmol L^-1^ | Linear range mmol L^-1^ | D  cm^2^s^-1^ | K_cat_  cm^3^mol^-1^s^-1^ | References |
| --- | --- | --- | --- | --- | --- |
| Fe_3_O_4_@C_r_/GCE  Fe_3_O_4_@C_s_/GCE  Fe_3_O_4_@C_d_/GCE  Fe_3_O_4_–GO/GCE  Co(OH)2NPs/GCE  Co/graphene/GCE  CoNPs/GCE  Ferrocene/carbon nanotube paste electrode  NWC nanotubes/gold nanorods/GCE  Acetaminophen/ruthenium oxide nanoparticles/GCE  Bi-powder carbon paste electrode | 2  8  26  25  220  0.89  0.41  0.49  0.00825  2.84  2 | 0.007-14.18  0.028-20.2  0.086-26.2  0.12-13.3  0.245-1.01  2.4-10.67  2.42-11.17  0.001-0.018  0.005-0.2  0.3-14  0.01-0.05 | 1.1×10^-6^  1.0×10^-6^  1.5×10^-6^  3.36×10^-5^  3.07×10^-6^  7.02×10^-5^  8.48×10^-6^  9.92×10^-6^  2.77×10^-6^  1.03×10^-6^  -- | 1.42×10^5^  9.63×10^4^  5.16×10^4^  1.24×10^5^  1.05×10^4^  1.03×10^6^  1.19×10^5^  3.07×10^5^  5.6×10^7^  --  -- | This work  This work  This work  [1]  [2]  [3]  [3]  [4]  [5]  [6]  [7] |

**Table S2.** Comparison of the performance of the three kinds of Fe_3_O_4_@C/GCE.

| Modified electrode | Detection limit  μmol L^−1^ | Linear range  mmol L^−1^ | D  cm^2^ s^-1^ | K_cat_  cm^3^ mol^-1^ s^-1^ |
| --- | --- | --- | --- | --- |
| Fe_3_O_4_@C_r_/GCE  Fe_3_O_4_@C_s_/GCE  Fe_3_O_4_@C_d_/GCE | 2  8  26 | 0.007-14.18  0.028-20.2,  0.086-26.2 | 1.10×10^-6^  1.09×10^-6^  1.50×10^-6^ | 1.42×10^5^  9.63×10^4^  5.16×10^4^ |

**Reference**

[1] Song, Y., He, Z., Hou, H., Wang, X. & Wang, L. Architecture of Fe_3_O_4_–graphene oxide nanocomposite and its application as a platform for amino acid biosensing. *Electrochim. Acta*. **71**, 58-65 (2012).

[2] Tabeshnia, M., et al. "Electrocatalytic oxidation of some amino acids on a cobalt hydroxide nanoparticles modified glassy carbon electrode." *Journal of Electroanalytical Chemistry* 647.2 (2010): 181-186.

[3] Song, Yonghai, et al. "Electrochemical and electrocatalytic properties of cobalt nanoparticles deposited on graphene modified glassy carbon electrode: Application to some amino acids detection." *Electrochimica Acta*58 (2011): 757-763.

[4] Raoof, Jahan Bakhsh, et al. "Carbon paste electrode incorporating multi-walled carbon nanotube/ferrocene as a sensor for the electroanalytical determination of N-acetyl-L-cysteine in the presence of tryptophan." *Journal of Chemical Sciences* 125.2 (2013): 283-289.

[5] dos Santos Silva, Francisco de Assis, et al. "A very low potential electrochemical detection of L-cysteine based on a glassy carbon electrode modified with multi-walled carbon nanotubes/gold nanorods." *Biosensors and Bioelectronics* 50 (2013): 202-209.

[6] Zare, Hamid R., and Fatemeh Chatraei. "Preparation and electrochemical characteristics of electrodeposited acetaminophen on ruthenium oxide nanoparticles and its role as a sensor for simultaneous determination of ascorbic acid, dopamine and N-acetyl-l-cysteine." *Sensors and Actuators B: Chemical* 160.1 (2011): 1450-1457.

[7] Baldrianova, L., et al. "The determination of cysteine at Bi-powder carbon paste electrodes by cathodic stripping voltammetry." *Electrochemistry Communications* 10.6 (2008): 918-921.

1. ^^ Corresponding author: Tel/Fax: +86 791 88120861. E-mail: [lwanggroup@aliyun.com](mailto:lwanggroup@aliyun.com) (L. Wang). [↑](#footnote-ref-2)
